# (3a*R*,8a*R*)-2,2,6,6-Tetra­methyl-4,4,8,8-tetra­phenyl­tetra­hydro-1,3-dioxolo[4,5-*e*][1,3,2]dioxasilepine

**DOI:** 10.1107/S160053680804227X

**Published:** 2008-12-17

**Authors:** Yousef M. Hijji, Paul F. Hudrlik, Anne M. Hudrlik, Ray J. Butcher, Jerry P. Jasinski

**Affiliations:** a Department of Chemistry, Morgan State University, Baltimore, MD 21251, USA; b Department of Chemistry, Howard University, 525 College Street NW, Washington, DC 20059, USA; c Department of Chemistry, Keene State College, 229 Main Street, Keene, NH 03435-2001, USA

## Abstract

The title compound, C_33_H_34_O_4_Si, is a dioxasilepine compound, an effective chiral dopant for the determination of high helical twisting powers in liquid crystals. Its structure consists of a five-membered dioxolo ring fused to a seven-membered dioxasilepine ring which contains two sets of phenyl rings in a twisted butterfly shape attached to the two C*sp* ^3^ atoms in the ring opposite each other. Two methyl groups are attached to the Si atom in the ring and two additional methyl groups are attached to the C*sp* ^3^ atom in the dioxolo ring (one of which is disordered) and which lies in an envelope pattern. The dihedral angles between the mean planes of the phenyl ring pairs are 85.9 (2) and 83.5 (1)°. The dihedral angles between the mean planes of the dioxolo ring and the two pairs of butterfly shaped phenyl rings are 46.2 (1), 67.7 (1), 35.6 (7) and 83.5 (1)°.

## Related literature

For a related structure, see: Madison *et al.* (1998 ▶). For dioxasilepines as chiral dopants in liquid crystals, see: Kuball & Hofer (2000 ▶); Kuball *et al.* (1997 ▶). For puckering parameters and pseudo rotation parameters, see: Cremer & Pople (1975 ▶); Rao *et al.* (1981 ▶).
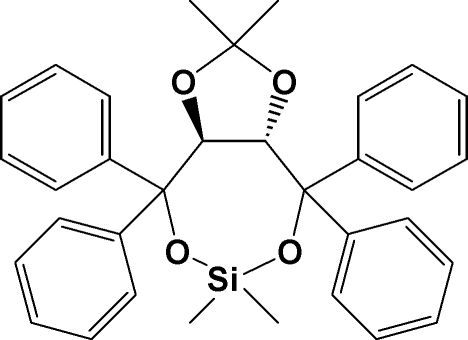


## Experimental

### 

#### Crystal data

C_33_H_34_O_4_Si
*M* *_r_* = 522.69
Orthorhombic, 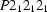

*a* = 10.008 (2) Å
*b* = 17.081 (3) Å
*c* = 17.271 (3) Å
*V* = 2952.4 (9) Å^3^
*Z* = 4
Mo *K*α radiation
μ = 0.11 mm^−1^
*T* = 293 (2) K
0.56 × 0.32 × 0.16 mm

#### Data collection

Siemans P2 diffractometer
Absorption correction: refined from Δ*F* (*SHELXL97*; Sheldrick, 2008 ▶) *T*_min_ = 0.786, *T*_max_ = 0.982
2819 measured reflections
2819 independent reflections
1693 reflections with *I* > 2σ(*I*)
3 standard reflections every 97 reflections intensity decay: none

#### Refinement

*R*[*F* ^2^ > 2σ(*F* ^2^)] = 0.054
*wR*(*F* ^2^) = 0.119
*S* = 1.04
2819 reflections
388 parameters
14 restraints
H-atom parameters constrained
Δρ_max_ = 0.24 e Å^−3^
Δρ_min_ = −0.18 e Å^−3^

### 

Data collection: *XSCANS* (Siemens, 2000 ▶); cell refinement: *XSCANS*; data reduction: *SHELXTL* (Sheldrick, 2008 ▶); program(s) used to solve structure: *SHELXS97* (Sheldrick, 2008 ▶); program(s) used to refine structure: *SHELXL97* (Sheldrick, 2008 ▶); molecular graphics: *SHELXTL*; software used to prepare material for publication: *SHELXTL*.

## Supplementary Material

Crystal structure: contains datablocks global, I. DOI: 10.1107/S160053680804227X/hg2455sup1.cif

Structure factors: contains datablocks I. DOI: 10.1107/S160053680804227X/hg2455Isup2.hkl

Additional supplementary materials:  crystallographic information; 3D view; checkCIF report

## Figures and Tables

**Fig. 1.**
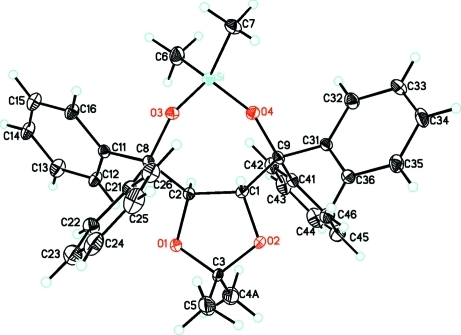
Molecular structure of the title compound, showing atom labeling and 50% probability displacement ellipsoids. Disordered aton C4A is shown with 0.38 (6) partial occupancy.

**Fig. 2.**
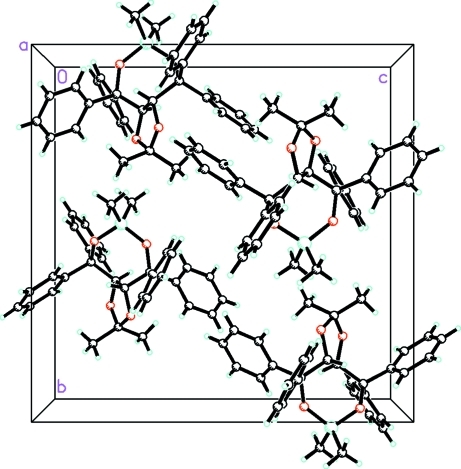
Packing diagram of the title compound, viewed down the a axis.

## supplementary crystallographic information

### Comment

Dioxasilepine compounds have been found to be effective chiral dopants for the determination of high helical twisting powers in liquid crystals (Kuball *et al.* 2000 and 1997). We have synthesized a new related structure and its crystal structure is reported.

The title compound, *C*_33_H_34_O_4_Si, consists of a 5-membered oxolo ring fused to a 7-membered dioxasilepine ring which contains two sets of phenyl rings in a twisted butterfly shape attached to the two *sp*^3^ carbon atoms in the ring opposite each other (Fig. 1). Two methyl groups are attached to the silicon atom in the ring and two additional methyl groups are attached to the *sp*^3^ carbon atom in the dioxolo ring (one of which is disordered at C4) which lies in an envelope pattern on C1 with pseudo rotation parameters P and Tau(*M*) of 172° and 18.5°, respectively (Rao *et al.*, 1981) for the refine bond C1—O2 [puckering parameters θ(2) = 0.1695 Å, Phi(2) = 80.2586° (Cremer & Pople, 1975)]. The dihedral angle between the mean planes of phenyl rings C11–C16 and C21–C26 is 85.9 (2)*^o^* and between phenyl rings C31–C36 and C41–C46 is 83.5 (1)*^o^*. Dihedreal angles between the mean planes of the dioxolo ring and the two butterfly shaped phenyl rings are 46.2 (1)*^o^* [C11–C16], 67.7 (1)*^o^* [C21–C26], 35.6 (7)*^o^* [C31–C36] and 83.5 (1)*^o^* [C41–C46], respectively.

Crystal packing is influenced by intermolecular C5A—H5A···*Cg*3 [3.628 (1) Å, *x, y, z*] and C4A—H4AA···*Cg*5 [3.757 (7) Å, *x, y, z*] π ring interactions where *Cg*3 and *Cg*5 = center of gravity of phenyl rings C21–C26 and C41–46, respectively (Fig. 2).

### Experimental

The title compound was synthesized by adding 0.13 g (1.06 mmol) of dimethylaminopyridine to a solution of 0.50 g (1.07 mmol) of (-)-*trans*-α,α'-(dimethyl-1,3-dioxolane-4,5-diyl)bis(diphenylmethanol) (TADDOL) in 20 ml of anhydrous ether (distilled from Na/benzophenone) under nitrogen atmosphere at room temperature. Then 0.145 g of imidazole (2.14 mmol) was added. The mixture was stirred to get a homogeneous solution. A solution of 0.138 g (1.07 mmol) dichlorodimethylsilane (distilled from CaH_2_) in 40 ml of anhydrous ether was added dropwise to the above solution. The mixture was stirred overnight under a nitrogen atmosphere. A white precipitate formed. The solid was filtered off through a sintered glass funnel under a blanket of nitrogen gas. Slow evaporation of the solvent under a stream of nitrogen gave white crystals (0.35 g; 62.5%). m.p 483–485 K. ^1^H NMR (CDCl_3_, CH_2_Cl_2_ standard, 300 MHz) δ 7.62, 7.60 (appears as d, J = 8 Hz, 4 H, Ar), 7.25–7.05 (m, 16 H, Ar), 5.15 (s, 2H, CH(OR), 0.52 (s, 6 H, CMe_2_), -0.25 (s, 6 H, SiMe_2_). ^13^C NMR (CDCl_3_, 75 MHz) δ 147.5, 143.1, 129.0, 127.9, 127.2, 127.1, 126.81, 126.76, 113.9, 82.2, 81.6, 27.0, -0.13.

### Refinement

The H atoms were placed in geometrically idealized positions and constrained to ride on their parent atoms with C—H distances in the range 0.93–0.98 Å and *U*_iso_(H) = 0.62–2.00*U*_eq_(C). The methyl carbon, C4, bonded to C1 is disordered with C4A at 0.38 (6) and C4B at 0.62 (6) partial occupancy. Since there was no atom present heavier that Si the absolute configuration could not be determined by X-ray methods. Hence the Friedel pairs were averaged.

### Crystal data

**Table:**

C_33_H_34_O_4_Si	*F*(000) = 1112
*M**_r_* = 522.69	*D*_x_ = 1.176 Mg m^−^^3^
Orthorhombic, *P*2_1_2_1_2_1_	Mo *K*α radiation, λ = 0.71073 Å
Hall symbol: P 2ac 2ab	Cell parameters from 26 reflections
*a* = 10.008 (2) Å	θ = 4.2–10.5°
*b* = 17.081 (3) Å	µ = 0.11 mm^−^^1^
*c* = 17.271 (3) Å	*T* = 293 K
*V* = 2952.4 (9) Å^3^	Block, colorless
*Z* = 4	0.56 × 0.32 × 0.16 mm

### Data collection

**Table:**

Siemans P2 diffractometer	1693 reflections with *I* > 2σ(*I*)
Radiation source: fine-focus sealed tube	θ_max_ = 25.0°, θ_min_ = 2.6°
graphite	*h* = 0→10
ω scans	*k* = 0→20
Absorption correction: part of the refinement model (Δ*F*) (*SHELXL97*; Sheldrick, 2008)	*l* = 0→20
*T*_min_ = 0.786, *T*_max_ = 0.982	3 standard reflections every 97 reflections
2819 measured reflections	intensity decay: none
2819 independent reflections	

### Refinement

**Table:**

Refinement on *F*^2^	Primary atom site location: structure-invariant direct methods
Least-squares matrix: full	Secondary atom site location: difference Fourier map
*R*[*F*^2^ > 2σ(*F*^2^)] = 0.054	Hydrogen site location: inferred from neighbouring sites
*wR*(*F*^2^) = 0.119	H-atom parameters constrained
*S* = 1.04	*w* = 1/[σ^2^(*F*_o_^2^) + (0.0282*P*)^2^ + 0.814*P*] where *P* = (*F*_o_^2^ + 2*F*_c_^2^)/3
2819 reflections	(Δ/σ)_max_ < 0.001
388 parameters	Δρ_max_ = 0.24 e Å^−^^3^
14 restraints	Δρ_min_ = −0.18 e Å^−^^3^

### Special details

**Table:**

Experimental. Sheldrick, G.M. (anon) SHELX97 Release 97-2 (1998) I/sigma threshold for reflections = 5.000 Delta(U)/lambda**2 = 0.000 Highest even order spherical harmonic = 6 Highest odd order spherical harmonic = 3
Geometry. All e.s.d.'s (except the e.s.d. in the dihedral angle between two l.s. planes) are estimated using the full covariance matrix. The cell e.s.d.'s are taken into account individually in the estimation of e.s.d.'s in distances, angles and torsion angles; correlations between e.s.d.'s in cell parameters are only used when they are defined by crystal symmetry. An approximate (isotropic) treatment of cell e.s.d.'s is used for estimating e.s.d.'s involving l.s. planes.
Refinement. Refinement of *F*^2^ against ALL reflections. The weighted *R*-factor *wR* and goodness of fit *S* are based on *F*^2^, conventional *R*-factors *R* are based on *F*, with *F* set to zero for negative *F*^2^. The threshold expression of *F*^2^ > σ(*F*^2^) is used only for calculating *R*-factors(gt) *etc*. and is not relevant to the choice of reflections for refinement. *R*-factors based on *F*^2^ are statistically about twice as large as those based on *F*, and *R*- factors based on ALL data will be even larger.

### Fractional atomic coordinates and isotropic or equivalent isotropic displacement parameters (Å^2^)

**Table:**

	*x*	*y*	*z*	*U*_iso_*/*U*_eq_	Occ. (<1)
Si	0.33141 (17)	0.47321 (9)	0.20896 (10)	0.0592 (5)	
O1	0.1516 (4)	0.7144 (2)	0.1711 (2)	0.0552 (10)	
O2	0.3460 (4)	0.73928 (18)	0.2371 (2)	0.0566 (10)	
O3	0.3023 (3)	0.5239 (2)	0.1311 (2)	0.0503 (9)	
O4	0.3509 (4)	0.53154 (19)	0.2825 (2)	0.0577 (10)	
C1	0.3489 (5)	0.6580 (3)	0.2198 (3)	0.0467 (14)	
H1A	0.4140	0.6486	0.1785	0.08 (2)*	
C2	0.2078 (5)	0.6398 (3)	0.1892 (3)	0.0451 (14)	
H2A	0.1554	0.6157	0.2308	0.038 (13)*	
C3	0.2373 (5)	0.7744 (3)	0.1978 (4)	0.0571 (16)	
C4A	0.1589 (18)	0.806 (2)	0.2655 (13)	0.112 (8)	0.52 (5)
H4AA	0.1480	0.7661	0.3038	0.168*	0.52 (5)
H4AB	0.0727	0.8236	0.2481	0.168*	0.52 (5)
H4AC	0.2063	0.8498	0.2878	0.168*	0.52 (5)
C4B	0.1677 (18)	0.8414 (12)	0.2382 (16)	0.069 (7)	0.48 (5)
H4BA	0.1374	0.8245	0.2882	0.103*	0.48 (5)
H4BB	0.0926	0.8580	0.2078	0.103*	0.48 (5)
H4BC	0.2289	0.8842	0.2442	0.103*	0.48 (5)
C5	0.2928 (8)	0.8209 (4)	0.1269 (5)	0.125 (3)	
H5A	0.3348	0.7853	0.0914	0.188*	
H5B	0.3572	0.8586	0.1445	0.188*	
H5C	0.2207	0.8474	0.1013	0.188*	
C6	0.1940 (9)	0.4087 (5)	0.2353 (5)	0.101 (3)	
H6A	0.1167	0.4395	0.2479	0.14 (4)*	
H6B	0.2191	0.3778	0.2793	0.16 (4)*	
H6C	0.1736	0.3748	0.1925	0.15 (4)*	
C7	0.4856 (8)	0.4183 (5)	0.1872 (5)	0.096 (3)	
H7A	0.5578	0.4544	0.1790	0.15 (4)*	
H7B	0.4726	0.3875	0.1413	0.16 (4)*	
H7C	0.5067	0.3845	0.2298	0.21 (5)*	
C8	0.2050 (5)	0.5850 (3)	0.1171 (3)	0.0446 (14)	
C9	0.3913 (5)	0.6119 (3)	0.2929 (3)	0.0475 (14)	
C11	0.0677 (5)	0.5469 (3)	0.1056 (3)	0.0460 (14)	
C12	−0.0482 (6)	0.5767 (4)	0.1375 (3)	0.0620 (16)	
H12A	−0.0448	0.6217	0.1677	0.036 (13)*	
C13	−0.1702 (7)	0.5395 (4)	0.1244 (4)	0.083 (2)	
H13A	−0.2479	0.5601	0.1458	0.063 (18)*	
C14	−0.1768 (8)	0.4733 (4)	0.0805 (4)	0.079 (2)	
H14A	−0.2585	0.4486	0.0724	0.12 (3)*	
C15	−0.0627 (7)	0.4432 (4)	0.0482 (4)	0.077 (2)	
H15A	−0.0665	0.3979	0.0184	0.12 (3)*	
C16	0.0574 (6)	0.4803 (4)	0.0602 (3)	0.0625 (16)	
H16A	0.1340	0.4601	0.0371	0.052 (16)*	
C21	0.2468 (6)	0.6290 (3)	0.0434 (3)	0.0508 (15)	
C22	0.1578 (8)	0.6732 (3)	0.0024 (4)	0.0698 (18)	
H22A	0.0691	0.6746	0.0183	0.07 (2)*	
C23	0.1945 (10)	0.7154 (5)	−0.0611 (4)	0.092 (2)	
H23A	0.1316	0.7452	−0.0876	0.16 (4)*	
C24	0.3253 (11)	0.7136 (5)	−0.0860 (4)	0.100 (3)	
H24A	0.3517	0.7429	−0.1287	0.11 (3)*	
C25	0.4167 (9)	0.6678 (5)	−0.0468 (5)	0.101 (3)	
H25A	0.5049	0.6658	−0.0637	0.13 (3)*	
C26	0.3781 (7)	0.6247 (4)	0.0176 (4)	0.070 (2)	
H26A	0.4397	0.5932	0.0432	0.09 (2)*	
C31	0.5427 (5)	0.6147 (3)	0.3048 (3)	0.0474 (14)	
C32	0.6025 (7)	0.5547 (4)	0.3465 (4)	0.0711 (19)	
H32A	0.5510	0.5132	0.3646	0.09 (2)*	
C33	0.7394 (7)	0.5563 (4)	0.3613 (4)	0.084 (2)	
H33A	0.7790	0.5154	0.3885	0.08 (2)*	
C34	0.8153 (7)	0.6169 (5)	0.3363 (4)	0.081 (2)	
H34A	0.9064	0.6180	0.3469	0.067 (18)*	
C35	0.7572 (6)	0.6772 (4)	0.2951 (4)	0.0707 (19)	
H35A	0.8095	0.7186	0.2776	0.07 (2)*	
C36	0.6220 (6)	0.6763 (3)	0.2797 (3)	0.0545 (15)	
H36A	0.5836	0.7173	0.2522	0.07 (2)*	
C41	0.3189 (6)	0.6414 (3)	0.3661 (3)	0.0532 (14)	
C42	0.2127 (6)	0.6020 (5)	0.3967 (4)	0.0684 (19)	
H42A	0.1834	0.5561	0.3731	0.07 (2)*	
C43	0.1473 (8)	0.6293 (6)	0.4625 (4)	0.094 (2)	
H43A	0.0743	0.6018	0.4818	0.10 (2)*	
C44	0.1888 (9)	0.6958 (6)	0.4992 (5)	0.103 (3)	
H44A	0.1450	0.7138	0.5432	0.13 (3)*	
C45	0.2967 (8)	0.7356 (5)	0.4695 (4)	0.089 (2)	
H45A	0.3262	0.7810	0.4939	0.08 (2)*	
C46	0.3619 (7)	0.7092 (4)	0.4038 (4)	0.0720 (18)	
H46A	0.4349	0.7368	0.3846	0.10 (3)*	

### Atomic displacement parameters (Å^2^)

**Table:**

	*U*^11^	*U*^22^	*U*^33^	*U*^12^	*U*^13^	*U*^23^
Si	0.0568 (10)	0.0456 (8)	0.0752 (12)	0.0000 (9)	−0.0127 (10)	0.0017 (10)
O1	0.048 (2)	0.050 (2)	0.067 (2)	0.003 (2)	−0.016 (2)	−0.0028 (19)
O2	0.062 (3)	0.0385 (19)	0.069 (3)	−0.006 (2)	−0.022 (2)	−0.0059 (19)
O3	0.051 (2)	0.0468 (19)	0.053 (2)	0.0058 (19)	0.0000 (18)	−0.003 (2)
O4	0.069 (2)	0.050 (2)	0.054 (2)	−0.007 (2)	−0.005 (2)	0.005 (2)
C1	0.051 (4)	0.042 (3)	0.047 (3)	−0.002 (3)	0.000 (3)	−0.006 (3)
C2	0.043 (3)	0.046 (3)	0.047 (4)	−0.003 (3)	−0.003 (3)	−0.001 (3)
C3	0.045 (3)	0.046 (3)	0.079 (5)	0.004 (3)	−0.026 (3)	−0.019 (4)
C4A	0.096 (9)	0.102 (12)	0.138 (11)	0.034 (9)	−0.031 (8)	−0.040 (9)
C4B	0.071 (8)	0.045 (8)	0.090 (11)	0.016 (7)	−0.015 (7)	−0.018 (7)
C5	0.133 (7)	0.096 (6)	0.147 (7)	−0.046 (6)	−0.065 (7)	0.059 (6)
C6	0.102 (7)	0.088 (5)	0.114 (7)	−0.041 (6)	−0.035 (5)	0.024 (6)
C7	0.095 (6)	0.089 (5)	0.105 (7)	0.045 (5)	−0.030 (5)	−0.024 (6)
C8	0.043 (3)	0.047 (3)	0.044 (3)	0.001 (3)	−0.007 (3)	−0.003 (3)
C9	0.046 (3)	0.053 (3)	0.043 (3)	0.001 (3)	−0.007 (3)	0.012 (3)
C11	0.047 (3)	0.050 (4)	0.041 (3)	−0.003 (3)	−0.002 (3)	0.006 (3)
C12	0.053 (4)	0.064 (4)	0.069 (4)	−0.013 (3)	0.004 (3)	−0.022 (4)
C13	0.048 (4)	0.107 (6)	0.094 (5)	−0.014 (4)	0.015 (4)	−0.020 (5)
C14	0.071 (5)	0.094 (5)	0.072 (5)	−0.030 (5)	0.005 (4)	−0.024 (4)
C15	0.074 (5)	0.087 (5)	0.070 (4)	−0.018 (4)	0.001 (4)	−0.023 (4)
C16	0.056 (4)	0.073 (4)	0.059 (4)	−0.002 (4)	0.008 (3)	−0.021 (4)
C21	0.054 (4)	0.052 (4)	0.046 (3)	−0.005 (3)	0.004 (3)	−0.010 (3)
C22	0.072 (5)	0.074 (4)	0.063 (4)	−0.011 (4)	0.003 (4)	0.017 (4)
C23	0.118 (7)	0.095 (5)	0.062 (5)	−0.018 (6)	0.000 (5)	0.017 (5)
C24	0.150 (9)	0.102 (6)	0.047 (5)	−0.054 (7)	0.009 (6)	0.003 (4)
C25	0.092 (7)	0.134 (8)	0.078 (6)	−0.037 (6)	0.031 (5)	−0.013 (6)
C26	0.061 (5)	0.086 (5)	0.064 (4)	−0.016 (4)	0.021 (4)	0.000 (4)
C31	0.048 (3)	0.048 (3)	0.047 (3)	0.009 (3)	−0.005 (3)	−0.005 (3)
C32	0.077 (5)	0.079 (5)	0.057 (4)	0.003 (4)	−0.011 (4)	0.011 (4)
C33	0.080 (6)	0.079 (5)	0.093 (6)	0.024 (5)	−0.028 (5)	0.008 (5)
C34	0.048 (5)	0.114 (6)	0.080 (5)	0.023 (5)	−0.017 (4)	−0.026 (5)
C35	0.041 (4)	0.089 (5)	0.082 (5)	−0.002 (4)	−0.001 (4)	−0.009 (5)
C36	0.048 (4)	0.055 (4)	0.060 (4)	0.004 (3)	−0.001 (3)	−0.001 (4)
C41	0.045 (3)	0.068 (4)	0.047 (4)	0.007 (3)	−0.002 (3)	0.003 (3)
C42	0.063 (5)	0.095 (5)	0.048 (4)	−0.002 (4)	0.007 (3)	0.005 (4)
C43	0.063 (5)	0.146 (8)	0.075 (6)	0.002 (6)	0.012 (5)	0.005 (6)
C44	0.076 (6)	0.154 (9)	0.078 (6)	0.026 (6)	0.026 (5)	−0.006 (6)
C45	0.112 (7)	0.096 (6)	0.060 (5)	0.020 (5)	−0.002 (5)	−0.031 (4)
C46	0.073 (5)	0.078 (4)	0.064 (4)	0.000 (4)	0.007 (4)	−0.013 (4)

### Geometric parameters (Å, °)

**Table:**

Si—O4	1.626 (4)	C13—C14	1.363 (8)
Si—O3	1.626 (4)	C13—H13A	0.9300
Si—C6	1.820 (7)	C14—C15	1.370 (9)
Si—C7	1.845 (7)	C14—H14A	0.9300
O1—C3	1.413 (6)	C15—C16	1.374 (8)
O1—C2	1.428 (6)	C15—H15A	0.9300
O2—C3	1.416 (6)	C16—H16A	0.9300
O2—C1	1.420 (5)	C21—C22	1.366 (8)
O3—C8	1.448 (6)	C21—C26	1.390 (8)
O4—C9	1.442 (6)	C22—C23	1.363 (9)
C1—C2	1.539 (7)	C22—H22A	0.9300
C1—C9	1.548 (6)	C23—C24	1.378 (12)
C1—H1A	0.9800	C23—H23A	0.9300
C2—C8	1.557 (7)	C24—C25	1.381 (11)
C2—H2A	0.9800	C24—H24A	0.9300
C3—C4B	1.511 (8)	C25—C26	1.387 (9)
C3—C4A	1.511 (9)	C25—H25A	0.9300
C3—C5	1.561 (9)	C26—H26A	0.9300
C4A—H4AA	0.9600	C31—C36	1.387 (7)
C4A—H4AB	0.9600	C31—C32	1.388 (7)
C4A—H4AC	0.9600	C32—C33	1.394 (9)
C4B—H4BA	0.9600	C32—H32A	0.9300
C4B—H4BB	0.9600	C33—C34	1.355 (9)
C4B—H4BC	0.9600	C33—H33A	0.9300
C5—H5A	0.9600	C34—C35	1.380 (9)
C5—H5B	0.9600	C34—H34A	0.9300
C5—H5C	0.9600	C35—C36	1.379 (8)
C6—H6A	0.9600	C35—H35A	0.9300
C6—H6B	0.9600	C36—H36A	0.9300
C6—H6C	0.9600	C41—C42	1.365 (8)
C7—H7A	0.9600	C41—C46	1.396 (8)
C7—H7B	0.9600	C42—C43	1.392 (9)
C7—H7C	0.9600	C42—H42A	0.9300
C8—C11	1.534 (7)	C43—C44	1.366 (10)
C8—C21	1.537 (7)	C43—H43A	0.9300
C9—C31	1.530 (7)	C44—C45	1.375 (10)
C9—C41	1.542 (7)	C44—H44A	0.9300
C11—C12	1.380 (7)	C45—C46	1.385 (9)
C11—C16	1.385 (7)	C45—H45A	0.9300
C12—C13	1.396 (8)	C46—H46A	0.9300
C12—H12A	0.9300		
			
O4—Si—O3	109.93 (17)	C12—C11—C8	123.0 (5)
O4—Si—C6	105.5 (4)	C16—C11—C8	119.2 (5)
O3—Si—C6	113.2 (3)	C11—C12—C13	120.2 (6)
O4—Si—C7	111.8 (3)	C11—C12—H12A	119.9
O3—Si—C7	104.6 (3)	C13—C12—H12A	119.9
C6—Si—C7	112.1 (5)	C14—C13—C12	120.7 (6)
C3—O1—C2	109.7 (4)	C14—C13—H13A	119.7
C3—O2—C1	109.1 (4)	C12—C13—H13A	119.7
C8—O3—Si	130.0 (3)	C13—C14—C15	119.8 (7)
C9—O4—Si	135.6 (3)	C13—C14—H14A	120.1
O2—C1—C2	104.6 (4)	C15—C14—H14A	120.1
O2—C1—C9	109.3 (4)	C14—C15—C16	119.7 (6)
C2—C1—C9	115.4 (4)	C14—C15—H15A	120.1
O2—C1—H1A	109.1	C16—C15—H15A	120.1
C2—C1—H1A	109.1	C15—C16—C11	121.9 (6)
C9—C1—H1A	109.1	C15—C16—H16A	119.1
O1—C2—C1	104.8 (4)	C11—C16—H16A	119.1
O1—C2—C8	110.7 (4)	C22—C21—C26	118.7 (6)
C1—C2—C8	114.4 (4)	C22—C21—C8	121.5 (5)
O1—C2—H2A	108.9	C26—C21—C8	119.9 (6)
C1—C2—H2A	108.9	C23—C22—C21	122.2 (8)
C8—C2—H2A	108.9	C23—C22—H22A	118.9
O1—C3—O2	108.4 (4)	C21—C22—H22A	118.9
O1—C3—C4B	114.8 (9)	C22—C23—C24	119.7 (9)
O2—C3—C4B	116.9 (10)	C22—C23—H23A	120.2
O1—C3—C4A	101.5 (12)	C24—C23—H23A	120.2
O2—C3—C4A	100.4 (13)	C23—C24—C25	119.3 (8)
C4B—C3—C4A	29.4 (9)	C23—C24—H24A	120.4
O1—C3—C5	109.3 (5)	C25—C24—H24A	120.4
O2—C3—C5	108.6 (5)	C24—C25—C26	120.6 (8)
C4B—C3—C5	98.0 (13)	C24—C25—H25A	119.7
C4A—C3—C5	127.4 (18)	C26—C25—H25A	119.7
C3—C4A—H4AA	109.5	C25—C26—C21	119.5 (8)
C3—C4A—H4AB	109.5	C25—C26—H26A	120.3
C3—C4A—H4AC	109.5	C21—C26—H26A	120.3
C3—C4B—H4BA	109.5	C36—C31—C32	118.4 (5)
C3—C4B—H4BB	109.5	C36—C31—C9	123.3 (5)
H4BA—C4B—H4BB	109.5	C32—C31—C9	118.3 (5)
C3—C4B—H4BC	109.5	C31—C32—C33	120.3 (7)
H4BA—C4B—H4BC	109.5	C31—C32—H32A	119.9
H4BB—C4B—H4BC	109.5	C33—C32—H32A	119.9
C3—C5—H5A	109.5	C34—C33—C32	120.5 (7)
C3—C5—H5B	109.5	C34—C33—H33A	119.7
H5A—C5—H5B	109.5	C32—C33—H33A	119.7
C3—C5—H5C	109.5	C33—C34—C35	119.9 (7)
H5A—C5—H5C	109.5	C33—C34—H34A	120.0
H5B—C5—H5C	109.5	C35—C34—H34A	120.0
Si—C6—H6A	109.5	C36—C35—C34	120.3 (7)
Si—C6—H6B	109.5	C36—C35—H35A	119.9
H6A—C6—H6B	109.5	C34—C35—H35A	119.9
Si—C6—H6C	109.5	C35—C36—C31	120.7 (6)
H6A—C6—H6C	109.5	C35—C36—H36A	119.7
H6B—C6—H6C	109.5	C31—C36—H36A	119.7
Si—C7—H7A	109.5	C42—C41—C46	118.0 (6)
Si—C7—H7B	109.5	C42—C41—C9	121.4 (6)
H7A—C7—H7B	109.5	C46—C41—C9	120.6 (5)
Si—C7—H7C	109.5	C41—C42—C43	121.1 (7)
H7A—C7—H7C	109.5	C41—C42—H42A	119.4
H7B—C7—H7C	109.5	C43—C42—H42A	119.4
O3—C8—C11	108.6 (4)	C44—C43—C42	121.0 (8)
O3—C8—C21	107.9 (4)	C44—C43—H43A	119.5
C11—C8—C21	110.1 (4)	C42—C43—H43A	119.5
O3—C8—C2	106.7 (4)	C43—C44—C45	118.5 (8)
C11—C8—C2	112.0 (4)	C43—C44—H44A	120.7
C21—C8—C2	111.4 (4)	C45—C44—H44A	120.7
O4—C9—C31	109.0 (4)	C44—C45—C46	120.9 (8)
O4—C9—C41	106.4 (4)	C44—C45—H45A	119.5
C31—C9—C41	110.1 (4)	C46—C45—H45A	119.5
O4—C9—C1	107.8 (4)	C45—C46—C41	120.5 (7)
C31—C9—C1	111.5 (4)	C45—C46—H46A	119.8
C41—C9—C1	111.9 (4)	C41—C46—H46A	119.8
C12—C11—C16	117.8 (5)		
			
O4—Si—O3—C8	−51.4 (4)	C11—C12—C13—C14	0.3 (10)
C6—Si—O3—C8	66.3 (5)	C12—C13—C14—C15	−0.6 (10)
C7—Si—O3—C8	−171.5 (5)	C13—C14—C15—C16	−0.3 (10)
O3—Si—O4—C9	−24.8 (5)	C14—C15—C16—C11	1.4 (10)
C6—Si—O4—C9	−147.2 (5)	C12—C11—C16—C15	−1.7 (8)
C7—Si—O4—C9	90.8 (6)	C8—C11—C16—C15	179.5 (6)
C3—O2—C1—C2	−18.7 (6)	O3—C8—C21—C22	160.2 (5)
C3—O2—C1—C9	−142.8 (4)	C11—C8—C21—C22	41.9 (7)
C3—O1—C2—C1	−8.0 (5)	C2—C8—C21—C22	−83.0 (6)
C3—O1—C2—C8	−131.9 (5)	O3—C8—C21—C26	−20.4 (7)
O2—C1—C2—O1	16.2 (5)	C11—C8—C21—C26	−138.7 (5)
C9—C1—C2—O1	136.3 (4)	C2—C8—C21—C26	96.4 (6)
O2—C1—C2—C8	137.6 (4)	C26—C21—C22—C23	−2.5 (9)
C9—C1—C2—C8	−102.2 (5)	C8—C21—C22—C23	177.0 (6)
C2—O1—C3—O2	−3.2 (6)	C21—C22—C23—C24	0.5 (10)
C2—O1—C3—C4B	−136.1 (14)	C22—C23—C24—C25	1.2 (12)
C2—O1—C3—C4A	−108.4 (16)	C23—C24—C25—C26	−0.8 (12)
C2—O1—C3—C5	114.9 (5)	C24—C25—C26—C21	−1.1 (11)
C1—O2—C3—O1	14.3 (6)	C22—C21—C26—C25	2.7 (9)
C1—O2—C3—C4B	146.1 (13)	C8—C21—C26—C25	−176.7 (6)
C1—O2—C3—C4A	120.3 (16)	O4—C9—C31—C36	147.2 (5)
C1—O2—C3—C5	−104.3 (5)	C41—C9—C31—C36	−96.5 (6)
Si—O3—C8—C11	−77.0 (5)	C1—C9—C31—C36	28.4 (7)
Si—O3—C8—C21	163.7 (3)	O4—C9—C31—C32	−36.3 (7)
Si—O3—C8—C2	43.9 (5)	C41—C9—C31—C32	80.0 (6)
O1—C2—C8—O3	160.3 (4)	C1—C9—C31—C32	−155.2 (5)
C1—C2—C8—O3	42.1 (5)	C36—C31—C32—C33	−1.0 (9)
O1—C2—C8—C11	−81.0 (5)	C9—C31—C32—C33	−177.6 (6)
C1—C2—C8—C11	160.8 (4)	C31—C32—C33—C34	1.1 (11)
O1—C2—C8—C21	42.8 (6)	C32—C33—C34—C35	−0.8 (11)
C1—C2—C8—C21	−75.3 (5)	C33—C34—C35—C36	0.5 (10)
Si—O4—C9—C31	−91.0 (5)	C34—C35—C36—C31	−0.5 (10)
Si—O4—C9—C41	150.3 (4)	C32—C31—C36—C35	0.7 (9)
Si—O4—C9—C1	30.1 (6)	C9—C31—C36—C35	177.2 (6)
O2—C1—C9—O4	161.0 (4)	O4—C9—C41—C42	−16.0 (6)
C2—C1—C9—O4	43.5 (6)	C31—C9—C41—C42	−133.9 (5)
O2—C1—C9—C31	−79.5 (5)	C1—C9—C41—C42	101.5 (6)
C2—C1—C9—C31	163.0 (4)	O4—C9—C41—C46	163.1 (5)
O2—C1—C9—C41	44.4 (6)	C31—C9—C41—C46	45.2 (7)
C2—C1—C9—C41	−73.1 (5)	C1—C9—C41—C46	−79.4 (6)
O3—C8—C11—C12	138.0 (5)	C46—C41—C42—C43	1.3 (9)
C21—C8—C11—C12	−104.1 (6)	C9—C41—C42—C43	−179.6 (6)
C2—C8—C11—C12	20.4 (7)	C41—C42—C43—C44	−0.9 (11)
O3—C8—C11—C16	−43.2 (6)	C42—C43—C44—C45	0.2 (12)
C21—C8—C11—C16	74.7 (6)	C43—C44—C45—C46	0.2 (11)
C2—C8—C11—C16	−160.8 (4)	C44—C45—C46—C41	0.2 (10)
C16—C11—C12—C13	0.8 (8)	C42—C41—C46—C45	−0.9 (9)
C8—C11—C12—C13	179.6 (6)	C9—C41—C46—C45	180.0 (6)

## References

[bb1] Cremer, D. & Pople, J. A. (1975). *J. Am. Chem. Soc.* **97**, 1254–1358.

[bb2] Kuball, H. G. & Hofer, T. (2000). *Chirality*, **12**, 278–286. 10.1002/(SICI)1520-636X(2000)12:4<278::AID-CHIR14>3.0.CO;2-O 10790198

[bb3] Kuball, H. G., Weiss, B., Beck, A. K. & Seebach, D. (1997). *Helv. Chim. Acta*, **80**, 2507–2514.

[bb4] Madison, J., Clausen, R. P., Hazell, R. G., Jacobson, H. J., Bols, M. & Perry, C. C. (1998). *Acta Chem. Scand.*, **52**, 1165–1170.

[bb5] Rao, S. T., Westhof, E. & Sundaralingam, M. (1981). *Acta Cryst.* A**37**, 421–425.

[bb6] Sheldrick, G. M. (2008). *Acta Cryst.* A**64**, 112–122. 10.1107/S0108767307043930 18156677

[bb7] Siemens (2000). *XSCANS* Bruker AXS Inc., Madison, Wisconsin, USA.

